# *Schistosoma japonicum*-derived peptide SJMHE1 ameliorates allergic symptoms and responses in mice with allergic rhinitis

**DOI:** 10.3389/fcimb.2023.1143950

**Published:** 2023-06-06

**Authors:** Xuerong Gao, Chaoming Mao, Tingting Zheng, Xiaowei Xu, Xinkai Luo, Shan Zhang, Jiameng Liu, Xuefeng Wang, Xiaojun Chen, Liyang Dong

**Affiliations:** ^1^ Department of Nuclear Medicine, The Affiliated Hospital of Jiangsu University, Zhenjiang, Jiangsu, China; ^2^ Department of Central Laboratory, The Affiliated Hospital of Jiangsu University, Zhenjiang, Jiangsu, China; ^3^ Department of Pathogen Biology & Immunology, Jiangsu Key Laboratory of Pathogen Biology, Nanjing Medical University, Nanjing, Jiangsu, China

**Keywords:** *Schistosoma japonicum* peptide SJMHE1, allergic rhinitis, spleen, CD19 cells, Bregs

## Abstract

Helminth derived excretory/secretory molecules have shown efficacy in the treatment of allergic asthma in mice, but their roles in allergic rhinitis (AR) are little known. In this study, we aimed to determine the intervention effect of SJMHE1, a *Schistosoma japonicum* derived small molecular peptide, on ovalbumin (OVA)-induced AR mice and investigate its possible mechanism. AR was induced in BALB/c mice, following which the mice were treated with phosphate-buffered saline (PBS), OVA^323-339^ and SJMHE1 respectively. SJMHE1 treatment improved clinical symptoms (rubbing and sneezing), suppressed infiltrates of inflammatory cells and eosinophils in nasal mucosa, modulated the production of type-2 (IL-4 and IL-13) and anti-inflammatory (IL-10) cytokines in the nasal lavage fluids (NLF), spleen, and serum. To investigate the underlying mechanism, fluorescein isothiocyanate (FITC)-labeled SJMHE1 was subcutaneously injected into AR mice, and we found that the FITC-SJMHE1 could accumulate in spleen, but not in nasal mucosa. FITC-SJMHE1 mainly bound to CD19 positive cells (B cells), and the SJMHE1 treatment significantly increased the proportion of regulatory B cells (Bregs) and B10 cells, along with the enhancement of PR domain containing protein 1 (Prdm1) protein levels. SJMHE1 may alleviate AR by upregulating Bregs, and has great potential as a new avenue for the AR treatment.

## Introduction

Allergic rhinitis (AR) is a common allergic inflammatory disease of the nasal airways, affecting 10%-40% of the global population, and having become a serious global public health concern (Brozek et al., 2017; Bousquet et al., 2020). Type-2 immune environment is the fundamental element of AR, which induces immunoglobulin E (IgE) produce and results in the appearance of symptoms such as rubbing and sneezing (Meng et al., 2019). Antihistamines, corticosteroids and antileukotrienes are effective for the symptom management of AR patients, however they cannot induce the disease regression, which prompts us to seek new therapeutic strategies.

Epidemiological studies exhibited an inverse geographic distribution of allergic inflammatory response and parasitic infections (Okada et al., 2010). Based on this, helminth therapy, especially helminth derived excretory/secretory molecules (helminth-E/S) (Harnett and Harnett, 2010), has rapidly been attempted as potential option to control dysregulated allergic responses. Indeed, some helminth-E/S, such as ES-62 (Rzepecka et al., 2014; Coltherd et al., 2016), HpARI (Osbourn et al., 2017), rSjP40 (Ren et al., 2016) and AIP-2 (Navarro et al., 2016), were reported to effectively prevent allergic airway inflammation in asthmatic mice, which confirmed that helminth-E/S are a new promising drugs for the treatment of allergic disease (Cruz et al., 2017; Bohnacker et al., 2020; Lothstein and Gause, 2021).

We have identified a peptide from the heat shock protein 60 (HSP60) of *Schistosoma japonicum* and named it SJMHE1 (Wang et al., 2009). In comparison with the above-mentioned helminth-E/S, SJMHE1 is a small molecule. SJMHE1 could suppress the inflammatory response in mice with ovalbumin (OVA)-induced delayed-type hypersensitivity (Wang et al., 2016), collagen-induced arthritis (Wang et al., 2017) and dextran sulfate sodium-induced colitis (Shan et al., 2021). Moreover, SJMHE1 ameliorated the airway inflammation of OVA-induced asthmatic mice and down regulated the expression of type 2-related cytokines (such as IL-4 and IL-13) (Zhang et al., 2019; Li et al., 2021). We hypothesized that SJMHE1 might be effective for the treatment of AR.

In this study, we explored the roles of SJMHE1 in AR mice and sought to determine its possible mechanisms.

## Materials and methods

### Peptides

Peptide SJMHE1 (VPGGGTALLRCIPVLDTLSTKNED) and its control OVA^323-339^ were synthesized and purified from Top-peptide (Shanghai, China). Polymyxin B-agarose was used to remove possible Lipopolysaccharide (LPS) contamination.

### Murine model of AR

Six week female BALB/c mice were purchased from the Comparative Medicine Centre of Yangzhou University (Yangzhou, China). Experimental AR was induced according to a previous report (Suzuki et al., 2020). Briefly, mice were sensitized with OVA (10 μg) and 2 mg of Al(OH)_3_ in 200 μl PBS intraperitoneally on days 0, 7 and 14. Subsequently, the mice were challenged intranasally with OVA (100 µg) on days 21–27 and 29-35 (once a day).

### Study design

**Figure 1:**
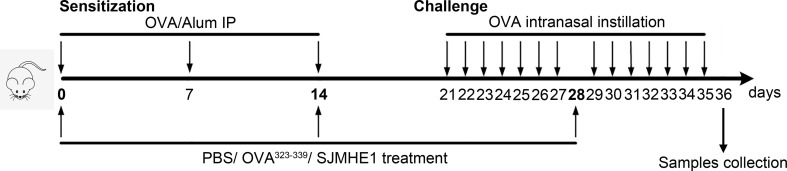
Experimental protocol for the development of allergic rhinitis and treatment with SJMHE1. BALB/c mice were sensitized with OVA and aluminum hydroxide in PBS on days 0, 7 and 14 by intraperitoneal injection (i.p.). Subsequently, the sensitized mice were challenged intranasally with OVA daily from days 21 to 27 and 29 to 35. PBS, emulsified OVA^323-339^ or SJMHE1 with incomplete Freund’s adjuvant were subcutaneously (inner thigh) injected into mice on days 0, 14 and 28. The mice were sacrificed on day 36 and the samples were collected.

Mice were randomly divided into four groups: Control, PBS, OVA^323-339^ and SJMHE1. Control group was normal healthy mouse. In groups PBS, OVA^323-339^ and SJMHE1, each mouse was sensitized with OVA (10 μg) and 2 mg of Al(OH)_3_ in 200 μl PBS intraperitoneally on days 0, 7 and 14, then, these mice were challenged intranasally with OVA (100 µg) on days 21–27 and 29-35. On days 0, 14 and 28, mice in the PBS, OVA^323-339^ and SJMHE1 groups were injected subcutaneously (inner thigh) with PBS (50 μL), OVA^323-339^ (10 µg; emulsified with incomplete Freund’s adjuvant) and SJMHE1 (10 µg; emulsified with incomplete Freund’s adjuvant), respectively. Mice were sacrificed at day-36, and the samples (such as nasal mucosa and serum) were collection. Study design was displayed in **Figure 1**.

### Nasal allergic symptoms

The number of nasal rubbing movements and sneezes was counted for 20 min immediately after the last OVA challenge on day 35.

### Histopathological analysis

All mice were sacrificed on day 36. The specimens of mice from each group were removed and immersed in 4% buffered paraformaldehyde, embedded in paraffin wax and sectioned at a thickness of 4 μm. Then, the paraffin-embedded sections were stained with hematoxylin and eosin (HE). Nasal mucosal smears have been used for morphological analysis (Pipkorn and Karlsson, 1988). The nasal mucosa was evenly smeared onto glass slides for Wright-Giemsa staining (BASO, Zhuhai, China).

### Nasal lavage fluids and serum cytokine detection

Levels of IL-4, IL-13 and IL-10 in the NLF and serum were measured using commercial enzyme-linked immune sorbent assay (ELISA) according to the manufacturer’s instructions (MultiSciences, Hangzhou, China).

### OVA-specific IgE measurement

The levels of OVA-specific IgE in mouse serum were carried out by ELISA in accordance with the method previously reported (Zhang et al., 2019; Li et al., 2021). The absorbance at 450 nm was detected with a microplate reader (Biotek Winooski, Vermont).

### Immunofluorescence

FITC-labeled SJMHE1 (10 µg; emulsified with incomplete Freund’s adjuvant) was injected into mice (inner thigh) subcutaneously (PBS was used as blank control). On day 3, 7, and 14, the OVA mice were sacrificed, the nasal mucosa and spleen were collected for frozen section. Finally, the sections were observed under an inverted fluorescence microscope (Nikon, Japan).

### Real-time PCR

Total RNA was extracted from the splenocytes of mice, and was reverse-transcribed into cDNA by using the Prime Script 1st Strand cDNA synthesis kit (Takara, Tokyo, Japan). Quantitative analysis of the relative mRNA expression was performed by using SYBR Premix Ex Taq (Takara) in a QuantStudio 5 Real-Time system (Termo Fisher Scientifc, Waltham, MA). All of the primers for real-time PCR were purchased from Tsingke Biotechnology (Nanjing, China). The average transcript levels of genes were normalized to β-actin. The relative mRNA expression was calculated using the formula 2^−△△Ct^ method, based on our previous description (Dong et al., 2014).

### Flow cytometry analysis

The fluorescent mAbs CD4, CD19, CD11c, F4/80, CD1d, CD5 and IL-10 were purchased from Elabscience (Wuhan, China). Intracellular staining was conducted as described previously (Yang et al., 2012). Splenocytes from mice were suspended in the presence of PMA/ionomycin mixture (Phorbol 12-myristate 13-acetate, Multisciences) and monensin (Multisciences) for 5 hours to analyze B10 cells (CD1d^hi^CD5^+^B19^+^IL-10^+^). Then, the cells were collected and stained with PE Anti-CD19 mAbs, FITC anti-CD1d mAbs and PerCP/Cyanine5.5 anti-CD5 mAbs. After removing the unbound antibodies, the cells were fixed and permeabilized with Cytofix/Cytoperm (BD Biosciences, San Jose, CA). Then, they were stained with APC mouse anti-IL-10 following the manufacturer’s instructions. The samples were performed using BD FACSCanto flow cytometer (BD Biosciences), and the results were analyzed using FlowJo Software (Version X; TreeStar, Ashland, OR).

### Western blot analysis

The proteins of the splenocytes in mice were extracted for Western Blot analysis as described previously (Dong et al., 2021). Anti-PRDM1/Blimp1 (abcam; 1:1000 dilution) and GAPDH (Proteintech, Wuhan, China; 1:1000 dilution) were used as the primary antibodies. HRP-conjugated ant-mouse IgG (Affinity Biosciences, Changzhou, China) was also utilized. ECL chemiluminescence kit (Thermo Fisher Scientific) was used for chemiluminescent detection followed by image analysis.

### RNA sequencing and analysis

1.5×10^6^ CD19^+^ cells from splenocytes were isolated by fluorescence-activated cell sorting (FACS). Total RNA samples extracted from the splenic B cells were subjected to RNA sequencing and analysis. The three samples from each group were mixed together separately for testing. RNA-seq experiment was carried out by LC-BIO Bio Technology (Hangzhou, China). After sequencing, the data were analyzed on the free online platform of LC-Bio Cloud Platform (https://www.omicstudio.cn/).

### Safety assessment of SJMHE1

Mice were treated with SJMHE1 every 2 weeks for 36 days (days 0, 14 and 28). Healthy mice were used as controls. Mice survival and body weight were recorded once a week. At day 36, mice serum were collected and the serum biochemistry including alanine aminotransferase (ALT), aspartate aminotransferase (AST), creatinine (CREA) and urea nitrogen (UREA) were measured using Beckman Coulter AU2700 automatic biochemical analyzer (Beckman Coulter, Miami, FL). Major organs (heart, liver, spleen, lungs and kidneys) of mice were collected for HE staining to assess histological changes.

### Statistical analyses

Statistical analyses were performed with GraphPad Prism 8.01 (GraphPad Software, San Diego, CA). The data are expressed as the mean ± standard deviation (SD). The groups were compared using the Student’s *t* test, one way ANOVA with Tukey Kramer *post hoc* tests. *P*<0.05 was considered statistically significant.

## Results

### SJMHE1 treatment reduced the numbers of rubbing movements and nasal sneezes in AR mice

**Figure 2:**
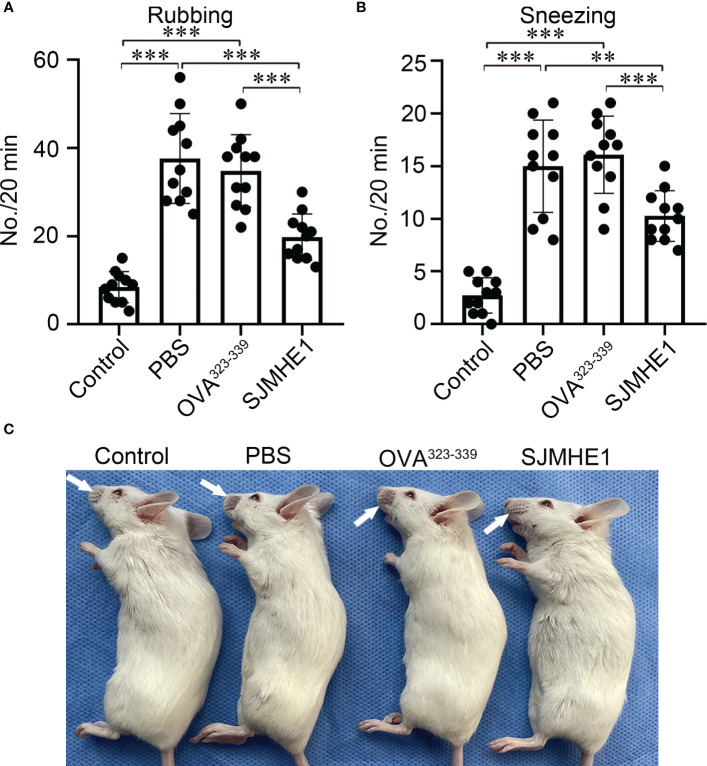
SJMHE1 treatment inhibits nasal rubbing and sneezes in allergic mice. The number of nasal rubbing **(A)** and sneezes movements **(B)** was counted for 20 min immediately after the last nasal challenge. Data are presented as mean ± SD (n = 11) from three independent experiments. Each dot represents data from one animal. **(C)** Representative image showing nasal external skin lesion induced by scratching. One-way analysis of variance (Tukey Kramer *post hoc* tests): **P<0.01, ***P<0.001.

AR mouse model was induced by OVA. PBS, OVA^323-339^ and SJMHE1 were subcutaneously injected into mice to examine the intervene effect (**Figure 1**). The number of rubbing movements and nasal sneezes were counted immediately after the last nasal challenge on day 35. Compared with control group (normal health mice), the OVA-challenged mice treated with PBS or OVA^323-339^ presented significantly increased number of rubbing movements and nasal sneezes. Compared with PBS or OVA^323-339^ group, SJMHE1 treatment dramatically reduced the nose-rubbing/sneezing events (**Figures 2A, B**), and inhibited OVA-induced skin denudation (**Figure 2C**).

### SJMHE1 attenuated inflammatory cell infiltration in nasal mucosa

**Figure 3:**
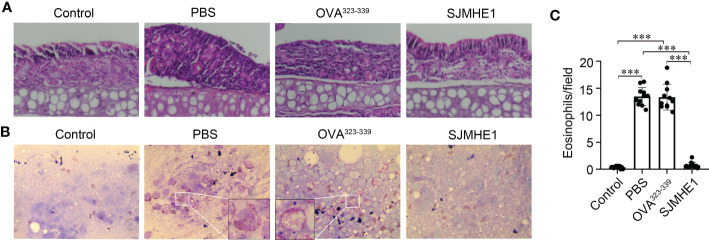
SJMHE1 treatment inhibits the development of nasal mucosa inflammation in allergic mice. **(A)** Representative photographs of HE-stained nasal mucosa sections from each group. 20× magnification. **(B)** Histological analysis of nasal mucosa from mice by Wright-Giemsa staining. 100× magnification. Representative images are showed. **(C)** The number of eosinophils in nasal mucosa was measured microscopically. Data are presented as mean ± SD (n = 11) from three independent experiments. Each dot represents data from one animal. One-way analysis of variance (Tukey Kramer *post hoc* tests): ***P<0.001.

The symptoms of AR are concentrated in the nose. Thus, the pathological changes of nasal mucosa in each group were investigated by using HE. As shown in **Figure 3A**, compared with control group, the PBS and OVA^323-339^ groups had a large amount of inflammatory cell infiltration. SJMHE1 treatment significantly reduced OVA-induced inflammatory cell infiltration. Next, the number of eosinophils in nasal mucosa was examined by using Wright-Giemsa staining. We found that SJMHE1 administration dramatically decreased eosinophils numbers, compared with PBS even or OVA^323-339^ treatments (**Figures 3B, C**).

### SJMHE1 treatment modulated cytokine expression in the NLF of AR mice

**Figure 4:**
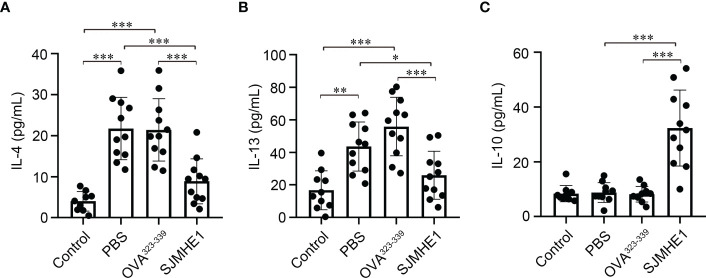
SJMHE1 treatment modulates the expression of cytokines in nasal lavage fluid (NLF) of allergic mice. On day 36, the mice were killed, and the NLF from each mouse was tested by ELISA. Statistical analysis of IL-4 **(A)**, IL-13 **(B)** and IL-10 **(C)** levels in the NLF. Data are presented as mean ± SD (n = 9-11) from three independent experiments. Each dot represents data from one animal. One-way analysis of variance (Tukey Kramer *post hoc* tests): *P<0.05, **P<0.01, ***P<0.001.

AR is accompanied by the release of type-2 cytokines, such as IL-4 and IL-13 (Eifan and Durham, 2016). As shown in **Figures 4A, B**, increased levels of IL-4 and IL-13 in NLF form PBS and OVA^323-339^ groups were observed. While, compared with PBS or OVA^323-339^ groups, treatment with SJMHE1 was able to significantly reduce the concentrations of IL-4 and IL-13. Then, the expression of IL-10, a famous anti-inflammatory factor that inhibits type 2 immune responses (Kosaka et al., 2011), was detected. No significant difference in IL-10 contents was found among the Control, PBS and OVA^323-339^ groups. SJMHE1 treatment upregulated the IL-10 levels compared with PBS and OVA^323-339^ groups (**Figure 4C**). Overall, the above results indicated that SJMHE1 could amend AR symptoms and reduce inflammatory responses in AR mice.

### SJMHE1 accumulated in spleen and regulate the systemic immune response in AR mice

**Figure 5:**
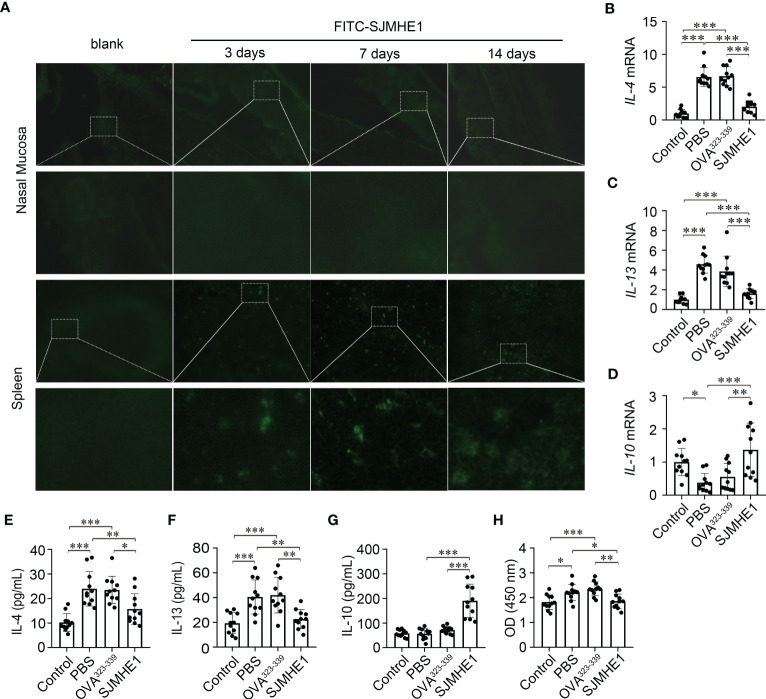
SJMHE1 accumulates in spleen and regulates the systemic immune response in AR mice. **(A)** Immunofluorescence of FITC-SJMHE1 (green) in nasal mucosa and spleen of mice at day 3, 7, and 14 after FITC-SJMHE1 treatment. Quantitative analysis for relative IL-4 **(B)**, IL-13 **(C)** and IL-10 **(D)** genes expression in the spleen of mice. Data are presented as mean ± SD (n = 11) from two independent experiments. Each dot represents data from one animal. **(E–G)** Statistical analysis of IL-4, IL-13 and IL-10 levels in the serum, respectively. Data are presented as mean ± SD (n = 11) from three independent experiments. Each dot represents data from one animal. **(H)** OVA-specific IgE level in serum of mice. Data are presented as mean ± SD (n = 11) from three independent experiments. Each dot represents data from one animal. One-way analysis of variance (Tukey Kramer *post hoc* tests): *P<0.05, **P<0.01, ***P<0.001.

To explore the mechanisms of SJMHE1-induced protection in AR, FITC-SJMHE (green) was injected into mice. First, the green signal in nasal mucosa was detected, as AR is a nasal airway inflammatory disease. While, no green fluorescence was observed at day 3, 7, and 14 post FITC-SJMHE1 injection. Given that SJMHE1 could regulate the spleen immune responses (Zhang et al., 2019; Li et al., 2021), we further scan the distribution of FITC-SJMHE1 in spleen. Indeed, green fluorescence was observed in mouse spleen at all inspection days (**Figure 5A**). Compared with the PBS group, the spleen mRNA levels of IL-4 and IL-13 in SJMHE1-treated mice were significantly down-regulated, and IL-10 expression was upregulated (**Figures 5B-D**). In addition, SJMHE1 treatment reduced the protein levels of IL-4 and IL-13 in serum, and increased the level of IL-10 (**Figures 5E-G**). Correspondingly, OVA-specific IgE levels in serum were significantly reduced after SJMHE1 treatment (**Figure 5H**).

### SJMHE1 mainly bound to B cells and increased the proportion of Bregs

**Figure 6:**
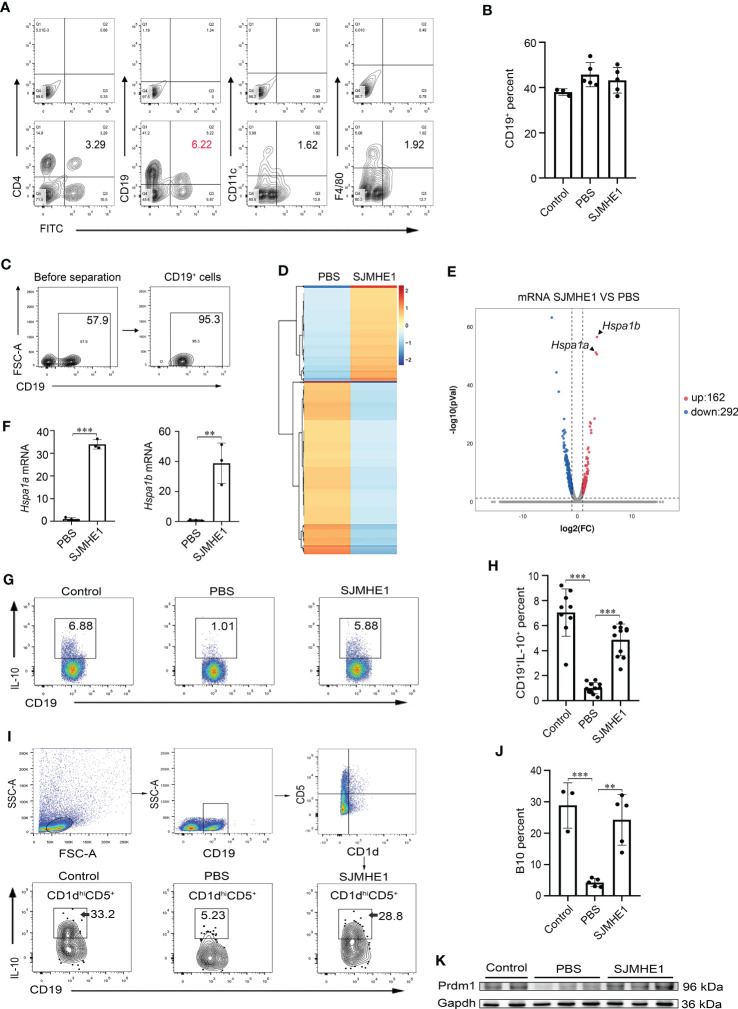
SJMHE1 binds to B cells in the spleen and upregulates the ratio of Bregs and B10 cells. **(A)** The percentages of CD4^+^FITC^+^ cells, CD19^+^FITC^+^ cells, CD11c^+^FITC^+^ cells and F4/80^+^FITC^+^ cells in spleen were analyzed by flow cytometry, and **(B)** percentage of CD19^+^ cells is shown (n = 3-5). **(C)** Splenocytes from mice were enriched for CD19^+^ cells using a flow cytometry sorter. **(D)** Gene expression profiles of the PBS and SJMHE1 groups were clustered in an unsupervised hierarchical manner. The heat map of differentially expressed genes identified by clustering analysis is shown in the figure. Each column represents a sample and each row represents a gene. Red and blue colors indicate genes with up- and down-regulated expression, respectively. **(E)** Dot plot shows 454 significantly differentially expressed (*P* < 0.05 and |log_2_FC| > 1) genes with 292 downregulated and 162 upregulated in the SJMHE1 group. FC: fold change. **(F)** Real-time PCR analysis of *Hspa1a* and *Hspa1b* mRNA levels in CD19^+^ B cells isolated from mouse splenocytes (n = 3). **(G, H)** Percentages of CD19^+^IL-10^+^ cells. Data are presented as mean ± SD (n = 9-11) from three independent experiments. Each dot represents data from one animal. **(I, J)** IL-10^+^ B-cell frequencies gated on CD1d^hi^CD5^+^ (n = 3-5). **(K)** Prmd1 expression in spleen of mice was detected by Western blotting (n = 2-3). One-way analysis of variance (Tukey Kramer *post hoc* tests) and Student’s *t* test: **P<0.01, ***P<0.001.

To identify the specific cell targeted by the SJMHE1, spleen cells were collected from AR mice post FITC-SJMHE1 injection 7 days and stained by using CD4 (T cells), CD19 (B cells), CD11c (DC cells), and F4/80 (macrophage). Intriguingly, flow cytometry showed that the percentage of CD19^+^FITC^+^ cells in the spleens were the highest in relative comparison to the CD4^+^FITC^+^, CD11c^+^FITC^+^, and F4/80^+^FITC^+^ (**Figure 6A**). Therefore, B cells were regarded as the focus of attention. Then, we analyzed the co-localization between FITC-SJMHE1 and B cells using fluorescence microscopy and found most FITC signal (green) co-localize with splenic CD19 cells (red), which further verified that SJMHE1could indeed directly bind with B cells (**Supplementary Figure 1**). B cells were not expanded upon SJMHE1 treatment compared with PBS group in AR mice (**Figure 6B**). To better understand the biological processes of SJMHE1 on B cells, spleen B cells were isolated from PBS and SJMHE1 groups by flow sorting method (**Figure 6C**), and then, a global RNA sequencing was performed. Data were analyzed based on Fragments Per Kilobase per Million mapped reads (FPKM), and the comparison generated a heat map and volcano plot of differentially expressed genes (**Figures 6D, E**). In total, SJMHE1 treatment showed 162 upregulated mRNAs and 292 downregulated mRNAs (**Additional file 1**). Interestingly, the most upregulated gene in above modules were *Hspa1a* and *Hspa1b* (**Figures 6E, F**), which encode heat shock protein 70 (HSP70) (Schroder et al., 2003) that are primarily expressed on Bregs (Wang et al., 2021). Bregs are immunosuppressive cells and they can inhibit immune response by producing IL-10 (Rosser and Mauri, 2015). Thus, the AR protection induced by SJMHE1 might involve in the upregulation of Bregs. Indeed, compare with the PBS group, the proportion of Bregs was increased in SJMHE1-treated mice (**Figures 6G, H**). Particularly, B10 cells (CD1d^hi^CD5^+^B19^+^IL-10^+^), a major Bregs subpopulation producing IL-10 (Xie et al., 2021), were also significantly increased by SJMHE1 administration (**Figures 6I, J**).

PR domain containing protein 1 (Prdm1) is considered as the key transcription factor for mediating Bregs differentiation (Wang et al., 2019b). Then, we detected its expression in spleen by Western blot analysis. As shown in **Figure 6K**, compared with PBS group, the protein levels of Prdm1 were enhanced in AR mice post-SJMHE1 administration.

**Figure 7:**
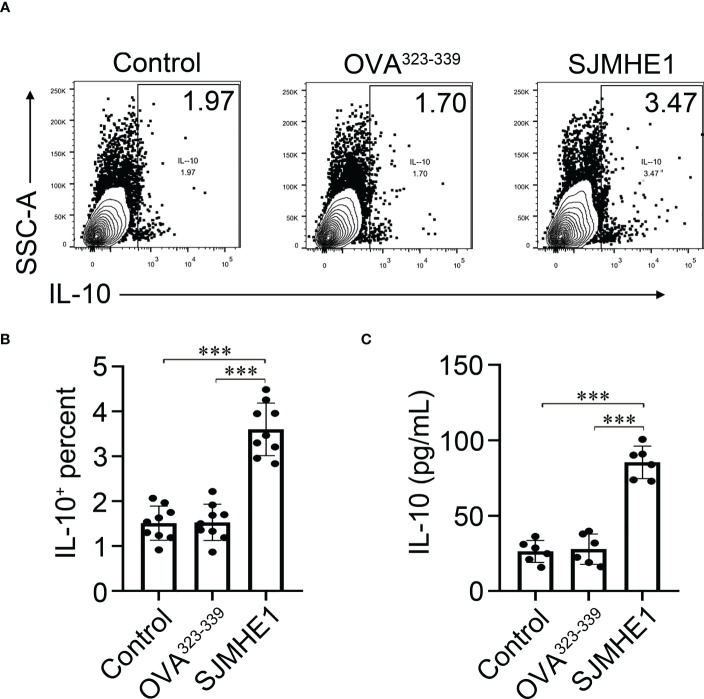
Breg cells can be generated *in vitro* by direct interaction with SJMHE1. **(A, B)** Splenic B cells from naïve mice were cultured for 24 h with SJMHE1 (0.5 μg/ml), OVA^323-339^ (0.5 μg/ml), or medium. Intracellular IL-10 expression of total B cells after addition of PMA/ionomycin and monenisn to the last 4 hours of the culture was determined by flow cytometry. Data are presented as mean ± SD (n = 9) from three independent experiments. **(C)** Splenic B cells from naïve mice were cultured with SJMHE1 (0.5 μg/ml) for 24 hours, and the protein levels of IL-10 in the supernatant were detected by ELISA. Data are presented as mean ± SD (n = 6) from two independent experiments. One-way analysis of variance (Tukey Kramer *post hoc* tests): ***P<0.001.

In addition, we performed an *in vitro* experiment where splenic B cells are exposed to the SJMHE1. Compared with control, SJMHE1 could significantly induce the numbers of IL-10 producing B cells (**Figures 7A, B**). In addition, a significantly higher levels of IL-10 were found in the supernatant of the B cell treated by SJMHE1 (**Figure 7C**).

### OVA-induced AR mice treated with SJMHE1 display changes in the pathway in the B cells

RNA-seq analysis on the spleen B cell were also performed comparing control group versus the SJMHE1 group, and control group versus PBS group. As shown in **Supplementary Figure 2**; **Additional files 2**, **3**, compared with control group, 1588 upregulated mRNAs and 1849 downregulated mRNAs was observed in SJMHE1 group, and 1662 upregulated mRNAs and 1804 downregulated mRNAs in PBS group. Kyoto Encyclopedia of Genes and Genomes (KEGG) was used to identify the biological pathways that were enriched most significantly in the above groups, and the top 20 most significantly enriched pathways included those related to lyfomome, ferroptosis, asthma, intestinal immune network for IgA production, and so on.

In addition, KEGG pathway analysis revealed that the genes that were most significantly differentially expressed between SJMHE1 and PBS groups were those associated with cytokine-cytokine receptor interactions, T cell receptor signaling pathway, JAK-STAT signaling pathway, MAPK signaling pathway, suggesting that SJMHE1 might drive the differentiation of B cells into Bregs through multiple targets.

### Toxicity of SJMHE1

**Figure 8:**
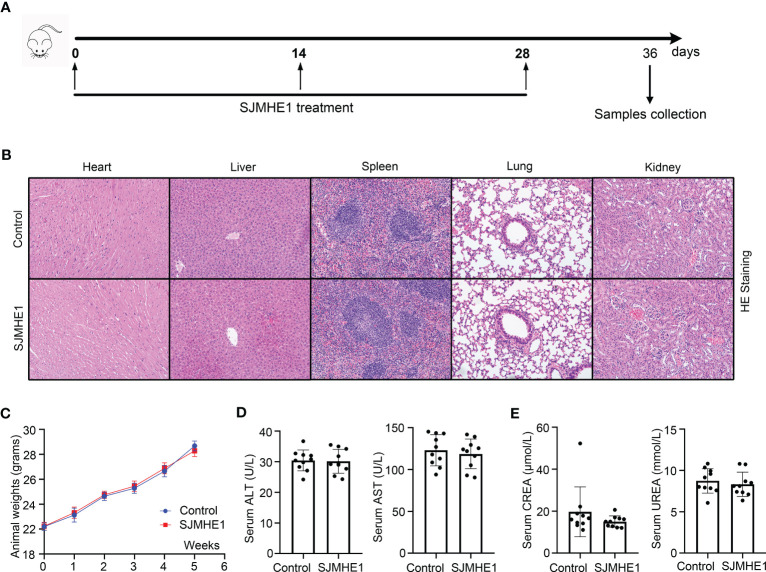
Biocompatibility evaluation of SJMHE1. **(A)** Schematic diagram for SJMHE1 treatment. **(B)** HE staining. **(C)** Body weight of mice. **(D, E)** The indicators reflecting the physiological functions of the liver (ALT and AST) and kidneys (CREA and UREA) were tested. Each dot represents data from one animal, and data are presented as mean ± SD (n = 10) from three independent experiments. Student’ s *t* test.

The safety of SJMHE1 treatment was evaluated during all *in vivo* studies (**Figure 8A**). No animal died in 36 days (date not shown). Compared with the health mice (Control group), no obvious tissue damages and inflammatory infiltration happened after SJMHE1 treatment in the HE staining of heart, liver, spleen, lung, and kidney (**Figure 8B**). SJMHE1 had no impact on mouse body weight (**Figure 8C**). ALT, AST, UREA, and CREA (the liver and kidney function-related blood biochemical values) in the serum of mice on day 36 were detected, and compared with the control, no significant differences have been found in the mice after SJMHE1 administration (**Figures 8D, E**).

## Discussion

AR is a widespread allergic disease and the ideal treatment strategies are still lacking. Recently, helminth-E/S are thought to be new promising therapeutics option for immunological disorders (Cruz et al., 2017; Bohnacker et al., 2020; Lothstein and Gause, 2021). Based on the previous findings that SJMHE1 was effective against airway inflammation in allergic asthma mice (Zhang et al., 2019; Li et al., 2021), we further explored the intervention effect of SJMHE1 on OVA-induced AR. To the best of our knowledge, this is the first report about the roles of helminth-E/S in AR.

OVA intraperitoneal injection followed by repeated upper-airway OVA challenge (intranasal) was used to induce allergic inflammation of the nasal mucosa, which is the well-established protocols of AR model (Suzuki et al., 2020; Fang et al., 2021). The nasal symptoms (eg, rubbing and sneezing) and antigen-specific IgE in serum play important roles in AR diagnosis (Greiner et al., 2011). Compared with PBS and OVA^323-339^, SJMHE1 effectively attenuated the allergic nasal symptoms, rubbing-caused nasal skin denudation, and OVA-specific IgE in OVA-induced AR mice, which was sufficient to demonstrate that SJMHE1 had an intervention effect on AR. In addition, compared with PBS, OVA^323-339^ did not exhibit intervention effect, partly suggesting that the SJMHE1’ effectiveness had certain specificity.

The nasal mucosa inflammation of AR is characterized by the eosinophils recruited to the nasal mucosa and the increased level of type-2 cytokines (eg, IL-4 and IL-13) (Meng et al., 2019). Our results showed that SJMHE1 was effective in alleviating nasal mucosa eosinophil infiltrations, accompanied by reduced protein levels of IL-4 and IL-13 in NALF. These findings were similar to previous reports that SJMHE1 possessed strong ability to inhibit local (lung) type-2 immune response in asthma animals (Zhang et al., 2019; Li et al., 2021). Furthermore, in this study, we first investigated the distribution of SJMHE1 in local (nasal mucosa) or peripheral (spleen) immune organ in AR mice, and found that SJMHE1 could accumulate in spleen, but not in the nasal mucosa. Added to the fact that compared with PBS and OVA^323-339^, SJMHE1 treatment significantly reduced the expression of IL-4 and IL-13 in AR mouse spleen and serum, and decreased the levels of OVA-specific IgE in serum, we considered that the intervention roles of the SJMHE1 in AR were mediated through the systemic immune response, indicating that SJMHE1 might also have intervention effect on other type-2 diseases.

The percentage of Bregs significantly decreased in the peripheral blood of AR patients (Kim et al., 2016; Wang and Tan, 2020) and in OVA-challenged AR mouse (Wang et al., 2019a). The subset of Bregs in peripheral blood was described to suppress the Th2 response through the secretion of IL-10 (Braza et al., 2014; Stanic et al., 2015). Bregs regulate the immune tolerance in AR and Bregs-based immunotherapy is in the making (Fan et al., 2022). Interestingly, Breg cells are considered relevant in helminth infection disease (van der Vlugt et al., 2014; Li et al., 2022), and during Schistosoma infection, active Breg cells were enriched in the spleen and suppressed allergic responses *via* elevated IL-10 production (Amu et al., 2010; van der Vlugt et al., 2012). Thus, whether SJMHE1 has a regulatory effect on Breg is worth exploring. In this study, we found that SJMHE1 (1) mostly bound to spleen B cells; (2) increased the proportion of Bregs and its major subpopulation B10 in AR mouse, together with the upregulation of *Hspa1a* and *Hspa1b* (encode HSP70 that are primarily expressed on Bregs); (3) enhanced the levels of IL-10; (4) upregulated the protein levels of Prdm1 in spleen; (5) could significantly induce the numbers of IL-10 producing B cells *in vitro*. Taken together, these data strongly confirmed that SJMHE1 had the ability to up-regulate the proportion of Bregs, which might partly explain its protective effect in AR. Although, we do not know how SJMHE1 regulate Bregs, it is still an interesting attempt to regard SJMHE1 as the agonist of Bregs and to treat other inflammatory disease.

In recent years, the therapeutic potential of SJMHE1 in several animal models has been verified (Lothstein and Gause, 2021), while, it is necessary to explore its toxicology. Compared with health mice, no significant changes in survival, body weight, pathological changes (including heart, liver, spleen, lungs and kidneys) and serum indices were found post SJMHE1 treatment, partially confirming the safety of SJMHE1 administration.

This study does have a number of limitations: (1) Different doses of SJMHE1 and different time points of SJMHE1 administration should be investigated, which are used to determine the optimal concentration and time of SJMHE1 for AR treatment. (2) Although the FITC-labeled SJMHE1 could be taken up by B cells, this effector mechanism is not clear.

In conclusions, SJMHE1 effectively reduced allergic symptoms and inflammatory parameters in the AR mouse, providing a potential therapeutic modality in AR.

## Data availability statement

The datasets presented in this study can be found in online repositories. The name of the repository and accession number(s) can be found below: NCBI Gene Expression Omnibus; accession number GSE229347.

## Ethics statement

The animal study was reviewed and approved by Institutional Animal Care and Use Committee of Jiangsu University.

## Author contributions

XG, CM, LD conceived and designed the experiments. XG, CM, TZ, XX, LD analyzed the data. XG, CM, TZ, XX, XL, SZ, JL, XW, XC, LD performed the experiments. The manuscript was written by XG, CM, LD. All authors read and approved the final manuscript.

## Supplementary material

Supplementary Figure 1 SJMHE1 bind to B cells. Immunofluorescence of FITC-SJMHE1 (green) in spleen of mice at day 3 after FITC-SJMHE1 treatment. Fluorescence microscopic image shows localization of FITC-SJMHE1 to CD19 B cells (indicated by the arrows). 10× magnification.

Supplementary Figure 2 OVA-induced AR mice treated with SJMHE1 display changes in the pathway in the B cells. **(A)** Volcano diagrams of mRNAs comparative gene expression analysis, and **(B)** differentially expressed genes were analyzed by KEGG enrichment analysis.

Additional file 1 RNA sequencing of spleen B cells. The SJMHE1 group had 454 differentially expressed genes compared to the PBS group.

Additional file 2 RNA sequencing of spleen B cells. The SJMHE1 group had 3437 differentially expressed genes compared to the Control group.

Additional file 3 RNA sequencing of spleen B cells. The PBS group had 3466 differentially expressed genes compared to the Control group.

## References

[B1] Amu S. Saunders S. P. Kronenberg M. Mangan N. E. Atzberger A. Fallon P. G. (2010). Regulatory b cells prevent and reverse allergic airway inflammation *via* FoxP3-positive T regulatory cells in a murine model. J. Allergy Clin. Immunol. 125 (5), 1114–1124.e1118. doi: 10.1016/j.jaci.2010.01.018 20304473

[B2] Bohnacker S. Troisi F. de Los Reyes Jimenez M. Esser-von Bieren J. (2020). What can parasites tell us about the pathogenesis and treatment of asthma and allergic diseases. Front. Immunol. 11. doi: 10.3389/fimmu.2020.02106 PMC7516051 33013887

[B3] Bousquet J. Anto J. M. Bachert C. Baiardini I. Bosnic-Anticevich S. Walter Canonica G.. (2020). Allergic rhinitis. Nat. Rev. Dis. Primers 6 (1), 95. doi: 10.1038/s41572-020-00227-0 33273461

[B4] Braza F. Chesne J. Castagnet S. Magnan A. Brouard S. (2014). Regulatory functions of b cells in allergic diseases. Allergy 69 (11), 1454–1463. doi: 10.1111/all.12490 25060230

[B5] Brozek J. L. Bousquet J. Agache I. Agarwal A. Bachert C. Bosnic-Anticevich S.. (2017). Allergic rhinitis and its impact on asthma (ARIA) guidelines-2016 revision. J. Allergy Clin. Immunol. 140 (4), 950–958. doi: 10.1016/j.jaci.2017.03.050 28602936

[B6] Coltherd J. C. Rodgers D. T. Lawrie R. E. Al-Riyami L. Suckling C. J. Harnett W.. (2016). The parasitic worm-derived immunomodulator, ES-62 and its drug-like small molecule analogues exhibit therapeutic potential in a model of chronic asthma. Sci. Rep. 6, 19224. doi: 10.1038/srep19224 26763929 PMC4725896

[B7] Cruz A. A. Cooper P. J. Figueiredo C. A. Alcantara-Neves N. M. Rodrigues L. C. Barreto M. L. (2017). Global issues in allergy and immunology: parasitic infections and allergy. J. Allergy Clin. Immunol. 140 (5), 1217–1228. doi: 10.1016/j.jaci.2017.09.005 29108604

[B8] Dong L. Wang X. Tan J. Li H. Qian W. Chen J.. (2014). Decreased expression of microRNA-21 correlates with the imbalance of Th17 and treg cells in patients with rheumatoid arthritis. J. Cell Mol. Med. 18 (11), 2213–2224. doi: 10.1111/jcmm.12353 25164131 PMC4224555

[B9] Dong L. Wang Y. Zheng T. Pu Y. Ma Y. Qi X.. (2021). Hypoxic hUCMSC-derived extracellular vesicles attenuate allergic airway inflammation and airway remodeling in chronic asthma mice. Stem Cell Res. Ther. 12 (1), 4. doi: 10.1186/s13287-020-02072-0 33407872 PMC7789736

[B10] Eifan A. O. Durham S. R. (2016). Pathogenesis of rhinitis. Clin. Exp. Allergy 46 (9), 1139–1151. doi: 10.1111/cea.12780 27434218

[B11] Fan K. Jin L. Yu S. (2022). Roles of regulatory b cells in the pathogenesis of allergic rhinitis. Allergol Immunopathol. (Madr) 50 (5), 7–15. doi: 10.15586/aei.v50i5.615 36086958

[B12] Fang Z. Yi F. Peng Y. Zhang J. J. Zhang L. Deng Z.. (2021). Inhibition of TRPA1 reduces airway inflammation and hyperresponsiveness in mice with allergic rhinitis. FASEB J. 35 (5), e21428. doi: 10.1096/fj.201902627R 33774861

[B13] Greiner A. N. Hellings P. W. Rotiroti G. Scadding G. K. (2011). Allergic rhinitis. Lancet 378 (9809), 2112–2122. doi: 10.1016/S0140-6736(11)60130-X 21783242

[B14] Harnett W. Harnett M. M. (2010). Helminth-derived immunomodulators: can understanding the worm produce the pill? Nat. Rev. Immunol. 10 (4), 278–284. doi: 10.1038/nri2730 20224568

[B15] Kim A. S. Doherty T. A. Karta M. R. Das S. Baum R. Rosenthal P.. (2016). Regulatory b cells and T follicular helper cells are reduced in allergic rhinitis. J. Allergy Clin. Immunol. 138 (4), 1192–1195.e1195. doi: 10.1016/j.jaci.2016.03.017 27142393 PMC5053844

[B16] Kosaka S. Tamauchi H. Terashima M. Maruyama H. Habu S. Kitasato H. (2011). IL-10 controls Th2-type cytokine production and eosinophil infiltration in a mouse model of allergic airway inflammation. Immunobiology 216 (7), 811–820. doi: 10.1016/j.imbio.2010.12.003 21257225

[B17] Li L. Shan W. Zhu H. Xue F. Ma Y. Dong L.. (2021). SJMHE1 peptide from schistosoma japonicum inhibits asthma in mice by regulating Th17/Treg cell balance *via* miR-155. J. Inflammation Res. 14, 5305–5318. doi: 10.2147/JIR.S334636 PMC8523811 34703270

[B18] Li M. Wang H. Ni Y. Li C. Xu X. Chang H.. (2022). Helminth-induced CD9(+) b-cell subset alleviates obesity-associated inflammation *via* IL-10 production. Int. J. Parasitol. 52 (2-3), 111–123. doi: 10.1016/j.ijpara.2021.08.009 34863801

[B19] Lothstein K. E. Gause W. C. (2021). Mining helminths for novel therapeutics. Trends Mol. Med. 27 (4), 345–364. doi: 10.1016/j.molmed.2020.12.010 33495068 PMC9884063

[B20] Meng Y. Wang C. Zhang L. (2019). Recent developments and highlights in allergic rhinitis. Allergy 74 (12), 2320–2328. doi: 10.1111/all.14067 31571226

[B21] Navarro S. Pickering D. A. Ferreira I. B. Jones L. Ryan S. Troy S.. (2016). Hookworm recombinant protein promotes regulatory T cell responses that suppress experimental asthma. Sci. Transl. Med. 8 (362), 362ra143. doi: 10.1126/scitranslmed.aaf8807 27797959

[B22] Okada H. Kuhn C. Feillet H. Bach J. F. (2010). The 'hygiene hypothesis' for autoimmune and allergic diseases: an update. Clin. Exp. Immunol. 160 (1), 1–9. doi: 10.1111/j.1365-2249.2010.04139.x PMC2841828 20415844

[B23] Osbourn M. Soares D. C. Vacca F. Cohen E. S. Scott I. C. Gregory W. F.. (2017). HpARI protein secreted by a helminth parasite suppresses interleukin-33. Immunity 47 (4), 739–751.e735. doi: 10.1016/j.immuni.2017.09.015 29045903 PMC5655542

[B24] Pipkorn U. Karlsson G. (1988). Methods for obtaining specimens from the nasal mucosa for morphological and biochemical analysis. Eur. Respir. J. 1 (9), 856–862. 3068074

[B25] Ren J. Hu L. Yang J. Yang L. Gao F. Lu P.. (2016). Novel T-cell epitopes on schistosoma japonicum SjP40 protein and their preventive effect on allergic asthma in mice. Eur. J. Immunol. 46 (5), 1203–1213. doi: 10.1002/eji.201545775 26840774

[B26] Rosser E. C. Mauri C. (2015). Regulatory b cells: origin, phenotype, and function. Immunity 42 (4), 607–612. doi: 10.1016/j.immuni.2015.04.005 25902480

[B27] Rzepecka J. Coates M. L. Saggar M. Al-Riyami L. Coltherd J. Tay H. K.. (2014). Small molecule analogues of the immunomodulatory parasitic helminth product ES-62 have anti-allergy properties. Int. J. Parasitol. 44 (9), 669–674. doi: 10.1016/j.ijpara.2014.05.001 24929132 PMC4119935

[B28] Schroder O. Schulte K. M. Ostermann P. Roher H. D. Ekkernkamp A. Laun R. A. (2003). Heat shock protein 70 genotypes HSPA1B and HSPA1L influence cytokine concentrations and interfere with outcome after major injury. Crit. Care Med. 31 (1), 73–79. doi: 10.1097/00003246-200301000-00011 12544996

[B29] Shan W. Zhang W. Xue F. Ma Y. Dong L. Wang T.. (2021). Schistosoma japonicum peptide SJMHE1 inhibits acute and chronic colitis induced by dextran sulfate sodium in mice. Parasit Vectors 14 (1), 455. doi: 10.1186/s13071-021-04977-y 34488863 PMC8422783

[B30] Stanic B. van de Veen W. Wirz O. F. Ruckert B. Morita H. Sollner S.. (2015). IL-10-overexpressing b cells regulate innate and adaptive immune responses. J. Allergy Clin. Immunol. 135 (3), 771–780.e778. doi: 10.1016/j.jaci.2014.07.041 25240783

[B31] Suzuki M. Yokota M. Kanemitsu Y. Min W. P. Ozaki S. Nakamura Y. (2020). Intranasal administration of regulatory dendritic cells is useful for the induction of nasal mucosal tolerance in a mice model of allergic rhinitis. World Allergy Organ J. 13 (8), 100447. doi: 10.1016/j.waojou.2020.100447 32817781 PMC7426451

[B32] van der Vlugt L. E. Labuda L. A. Ozir-Fazalalikhan A. Lievers E. Gloudemans A. K. Liu K. Y.. (2012). Schistosomes induce regulatory features in human and mouse CD1d(hi) b cells: inhibition of allergic inflammation by IL-10 and regulatory T cells. PLoS One 7 (2), e30883. doi: 10.1371/journal.pone.0030883 22347409 PMC3275567

[B33] van der Vlugt L. E. Zinsou J. F. Ozir-Fazalalikhan A. Kremsner P. G. Yazdanbakhsh M. Adegnika A. A.. (2014). Interleukin 10 (IL-10)-producing CD1dhi regulatory b cells from schistosoma haematobium-infected individuals induce IL-10-positive T cells and suppress effector T-cell cytokines. J. Infect. Dis. 210 (8), 1207–1216. doi: 10.1093/infdis/jiu257 24795476

[B34] Wang L. Fu Y. Yu B. Jiang X. Liu H. Liu J.. (2021). HSP70, a novel regulatory molecule in b cell-mediated suppression of autoimmune diseases. J. Mol. Biol. 433 (1), 166634. doi: 10.1016/j.jmb.2020.08.019 32860772

[B35] Wang M. Gu Z. Yang J. Zhao H. Cao Z. (2019a). Changes among TGF-beta1(+) breg cells and helper T cell subsets in a murine model of allergic rhinitis with prolonged OVA challenge. Int. Immunopharmacol 69, 347–357. doi: 10.1016/j.intimp.2019.01.009 30776643

[B36] Wang X. Li L. Wang J. Dong L. Shu Y. Liang Y.. (2017). Inhibition of cytokine response to TLR stimulation and alleviation of collagen-induced arthritis in mice by schistosoma japonicum peptide SJMHE1. J. Cell Mol. Med. 21 (3), 475–486. doi: 10.1111/jcmm.12991 27677654 PMC5323857

[B37] Wang Z. Tan F. (2020). The blockade of PD-1/PD-L1 pathway promotes the apoptosis of CD19(+) CD25(+) bregs and suppresses the secretion of IL-10 in patients with allergic rhinitis. Scand. J. Immunol. 91 (2), e12836. doi: 10.1111/sji.12836 31598989

[B38] Wang Y. H. Tsai D. Y. Ko Y. A. Yang T. T. Lin I. Y. Hung K. H.. (2019b). Blimp-1 contributes to the development and function of regulatory b cells. Front. Immunol. 10. doi: 10.3389/fimmu.2019.01909 PMC6702260 31474988

[B39] Wang X. Wang J. Liang Y. Ni H. Shi L. Xu C.. (2016). Schistosoma japonicum HSP60-derived peptide SJMHE1 suppresses delayed-type hypersensitivity in a murine model. Parasit Vectors 9, 147. doi: 10.1186/s13071-016-1434-4 26971312 PMC4789290

[B40] Wang X. Zhou S. Chi Y. Wen X. Hoellwarth J. He L.. (2009). CD4+CD25+ treg induction by an HSP60-derived peptide SJMHE1 from schistosoma japonicum is TLR2 dependent. Eur. J. Immunol. 39 (11), 3052–3065. doi: 10.1002/eji.200939335 19882655

[B41] Xie J. Shi C. W. Huang H. B. Yang W. T. Jiang Y. L. Ye L. P.. (2021). Induction of the IL-10-producing regulatory b cell phenotype following trichinella spiralis infection. Mol. Immunol. 133, 86–94. doi: 10.1016/j.molimm.2021.02.012 33636433

[B42] Yang M. Deng J. Liu Y. Ko K. H. Wang X. Jiao Z.. (2012). IL-10-producing regulatory B10 cells ameliorate collagen-induced arthritis *via* suppressing Th17 cell generation. Am. J. Pathol. 180 (6), 2375–2385. doi: 10.1016/j.ajpath.2012.03.010 22538089

[B43] Zhang W. Li L. Zheng Y. Xue F. Yu M. Ma Y.. (2019). Schistosoma japonicum peptide SJMHE1 suppresses airway inflammation of allergic asthma in mice. J. Cell Mol. Med. 23 (11), 7819–7829. doi: 10.1111/jcmm.14661 31496071 PMC6815837

