# Supplementary figures and images for: Schistosoma japonicum-derived peptide SJMHE1 ameliorates allergic symptoms and responses in mice with allergic rhinitis
Source: Front Cell Infect Microbiol. 2023 Jun 6;13:1143950. doi: 10.3389/fcimb.2023.1143950 (PMC10279851; doi:10.3389/fcimb.2023.1143950)

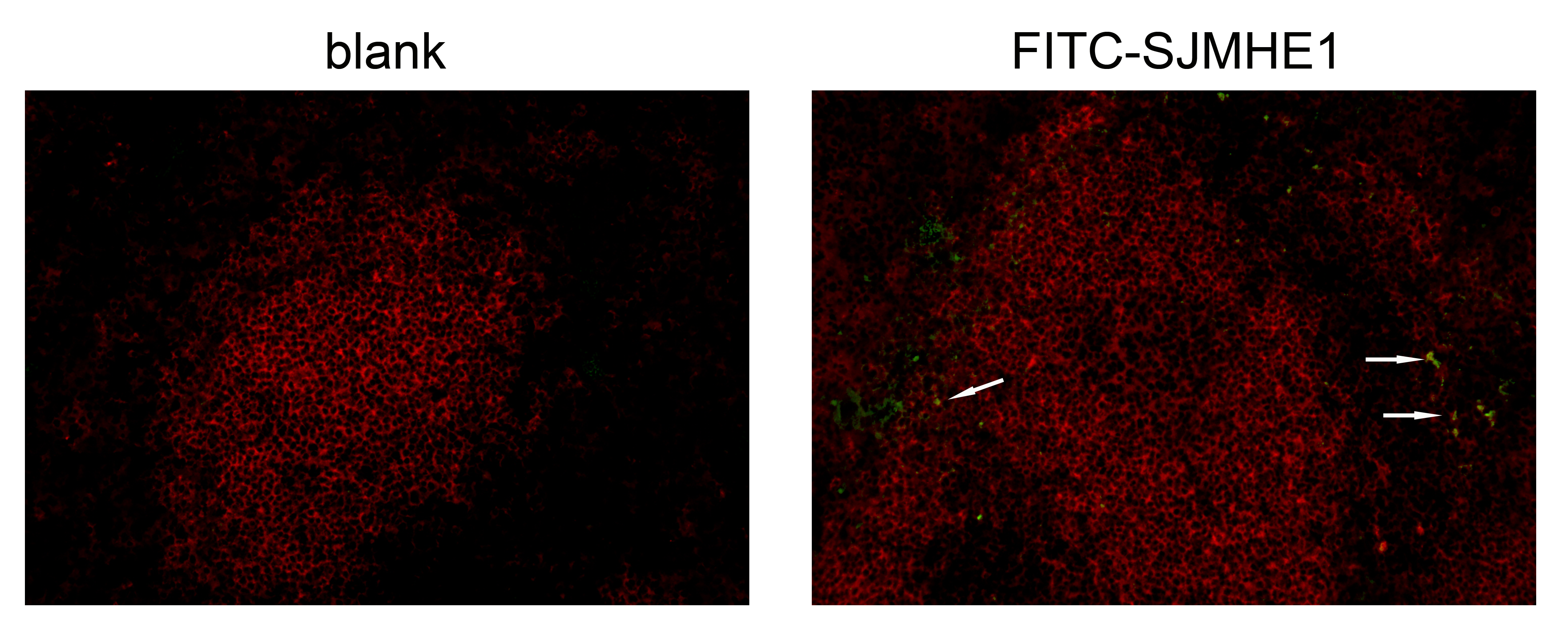

Supplement: Supplementary Figure 1 — SJMHE1 bind to B cells. Immunofluorescence of FITC-SJMHE1 (green) in spleen of mice at day 3 after FITC-SJMHE1 treatment. Fluorescence microscopic image shows localization of FITC-SJMHE1 to CD19 B cells (indicated by the arrows). 10× magnification. [file Image_1.tif]

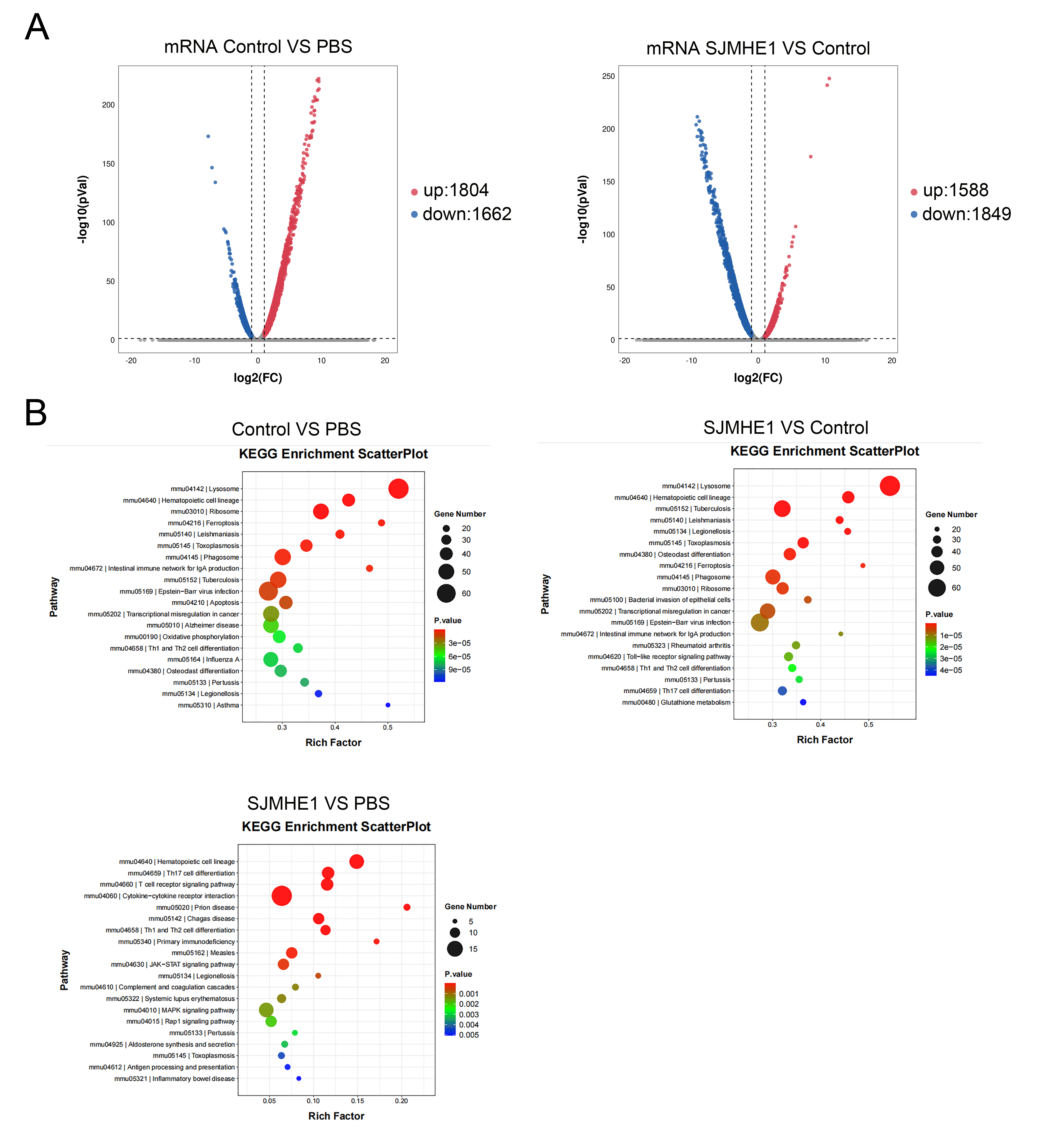

Supplement: Supplementary Figure 2 — OVA-induced AR mice treated with SJMHE1 display changes in the pathway in the B cells. (A) Volcano diagrams of mRNAs comparative gene expression analysis, and (B) differentially expressed genes were analyzed by KEGG enrichment analysis. [file Image_2.tif]
